# Sodium [^18^F]Fluoride PET Can Efficiently Monitor In Vivo Atherosclerotic Plaque Calcification Progression and Treatment

**DOI:** 10.3390/cells10020275

**Published:** 2021-01-30

**Authors:** Alexandru Florea, Julius P. Sigl, Agnieszka Morgenroth, Andreas Vogg, Sabri Sahnoun, Oliver H. Winz, Jan Bucerius, Leon J. Schurgers, Felix M. Mottaghy

**Affiliations:** 1 Department of Nuclear Medicine, University Hospital RWTH Aachen, 52074 Aachen, Germany; aflorea@ukaachen.de (A.F.); julius.sigl@googlemail.com (J.P.S.); amorgenroth@ukaachen.de (A.M.); avogg@ukaachen.de (A.V.); ssahnoun@ukaachen.de (S.S.); owinz@ukaachen.de (O.H.W.); 2 Department of Radiology and Nuclear Medicine, Maastricht University Medical Center, 6229 HX Maastricht, The Netherlands; jan.bucerius@med.uni-goettingen.de; 3 School for Cardiovascular Diseases (CARIM), Maastricht University Medical Center, 6229 HX Maastricht, The Netherlands; l.schurgers@maastrichtuniversity.nl; 4 Department of Nuclear Medicine, University of Göttingen, 37075 Göttingen, Germany; 5 Department of Biochemistry, Maastricht University Medical Center, 6229 HX Maastricht, The Netherlands; 6 Institute of Experimental Medicine and Systems Biology, RWTH Aachen University, 52074 Aachen, Germany

**Keywords:** cardiovascular diseases, positron-emission tomography, sodium fluoride, vitamin K, warfarin, vascular calcification, micro-calcification

## Abstract

Given the high sensitivity and specificity of sodium [^18^F]Fluoride (Na[^18^F]F) for vascular calcifications and positive emerging data of vitamin K on vascular health, the aim of this study is to assess the ability of Na[^18^F]F to monitor therapy and disease progression in a unitary atherosclerotic mouse model. ApoE^−/−^ mice were placed on a Western-type diet for 12-weeks and then split into four groups. The early stage atherosclerosis group received a chow diet for an additional 12-weeks, while the advanced atherosclerosis group continued the Western-type diet. The Menaquinone-7 (MK-7) and Warfarin groups received MK-7 or Warfarin supplementation during the additional 12-weeks, respectively. Control wild type mice were fed a chow diet for 24-weeks. All of the mice were scanned with Na[^18^F]F using a small animal positron emission tomography (PET)/computed tomography (CT). The Warfarin group presented spotty calcifications on the CT in the proximal aorta. All of the spots corresponded to dense mineralisations on the von Kossa staining. After the control, the MK-7 group had the lowest Na[^18^F]F uptake. The advanced and Warfarin groups presented the highest uptake in the aortic arch and left ventricle. The advanced stage group did not develop spotty calcifications, however Na[^18^F]F uptake was still observed, suggesting the presence of micro-calcifications. In a newly applied mouse model, developing spotty calcifications on CT exclusively in the proximal aorta, Na[^18^F]F seems to efficiently monitor plaque progression and the beneficial effects of vitamin K on cardiovascular disease.

## 1. Introduction

Medical associations around the globe, including the American Heart Association, recommend the early identification of subclinical atherosclerosis via non-invasive imaging (i.e., coronary artery calcification score or carotid artery ultrasound) in order to guide preventive care [1]. Indeed, considering the recent advancements in medical imaging, these techniques can be used for patient selection and stratification [2].

Numerous imaging techniques have been proposed for the identification of vascular calcification, especially after its acknowledgment as an independent cardiovascular risk factor and as a marker for plaque vulnerability, alongside inflammation [3,4]. In a recent review, the superiority of sodium [^18^F]Fluoride (Na[^18^F]F) positron emission tomography (PET) over other modalities to correctly identify micro-calcified plaque has been elaborated [5]. Na[^18^F]F is a PET tracer that binds to hydroxyapatite crystals and is therefore able to identify osseous and ectopic calcifications (including vascular calcifications). Moreover, its high specificity makes it highly valuable for the non-invasive identification of micro-calcified plaque [6]. It is also considered that Na[^18^F]F uptake in plaque with no visible calcifications on the computed tomography (CT) is a result of actively developing micro-calcifications [7]. The smallest vascular calcification formation visible with computed tomography (CT) is termed “spotty calcification”, being less than 3 mm in diameter and having an increased attenuation compared with the surrounding tissue [8]. However, the Multi-Ethnic Study of Atherosclerosis study (i.e., MESA) showed an inverse association of vascular calcification on CTs with cardiovascular risk [9]. Na[^18^F]F PET remains the only available clinical tool that can non-invasively detect potentially vulnerable micro-calcified plaque [5,10].

Given the high sensitivity and specificity of Na[^18^F]F for vascular calcifications, we wanted to investigate the ability of this tracer to monitor therapy and disease progression. In a recent review paper, we also presented emerging data that suggest vitamin K—especially MK-7—as a cost-effective method for delaying the progression of vascular calcification [5]. In short, vitamin K is a crucial cofactor in the post-translational modification of various proteins, including coagulation factors and matrix γ-carboxyglutamate protein (i.e., MGP), the latter of which is involved in the inhibition of ectopic calcification. Considering this mechanism of action, vitamin K has received the attention of several on-going clinical trials. Out of all vitamins that fall under the umbrella of “vitamin K”, MK-7 is one of the most used in preclinical and clinical trials, thanks to its increased half-life in blood, which extends its uptake availability through extra-hepatic tissues [11]. Conversely, the vitamin K inhibitor Warfarin is able to block the post-translational modifications of the matrix γ-carboxyglutamate protein, and thus accelerates ectopic calcifications, including vascular ones. It is well known that rodents under Warfarin treatment alone develop lethal bleedings caused by inhibiting the proper hepatic synthesis of vitamin K-dependent coagulation factors. By supplementing the respective diets with Phylloquinone (i.e., vitamin K1), the liver function is recovered; however, Warfarin is still able to inhibit the proper synthesis of matrix γ-carboxyglutamate protein (i.e., MGP) [12,13,14].

In our current study, we wanted to assess the ability of Na[^18^F]F to monitor therapy and disease progression in a unitary atherosclerotic mouse model. Hence, the ability of Na[^18^F]F PET to detect small changes in plaque morphology was prospectively tested in atherosclerotic mice, that were given either Menaquinone-7 (MK-7) supplementation, or Warfarin or Phylloquinone supplementation, or were maintained on the atherogenic diet.

## 2. Materials and Methods

### 2.1. Mouse Strains and Care

All of the animal experiments were approved by a German competent authority (Landesamt für Natur, Umwelt und Verbraucherschutz Nordrhein-Westfalen) for compliance with the Animal Protection Act, in conjunction with the regulation for the protection of animals used for experimental and other scientific purposes (file number 81-02.04.2018.A286). Wild type (*n* = 5) and ApoE^−/−^ (*n* = 20) male mice were purchased from Charles River Laboratories Italy (i.e., C57BL/6J and B6.129P2-Apoe^tm1Unc/J^, respectively). Upon arrival, all of the mice were kept in the Institute for Laboratory Animal Science of the University Hospital RWTH Aachen, and one week prior to the scans, they were moved to the Department of Nuclear medicine of the University Hospital RWTH Aachen for acclimatization.

The animals were housed under a 12-h-light/12-h-dark cycle and were given free access to their respective diets and to water. The room temperature and relative humidity were kept between 20–25 °C and 45–65%, respectively.

Because all of mice used had a C57BL/6 background, and in combination of the 24-weeks feeding scheme, some animals developed skin lesions suggestive of ulcerative dermatitis. Hence, a Dexpanthenol ointment (Bepanthen^®^ from Bayer Vital GmbH, Leverkusen, Germany) was applied to ease scratching; moreover, these mice had priority on the day of scanning.

Over the course of the study, one animal was lost due to anesthesia.

### 2.2. Experimental Groups and Feeding Scheme

**Figure 1:**
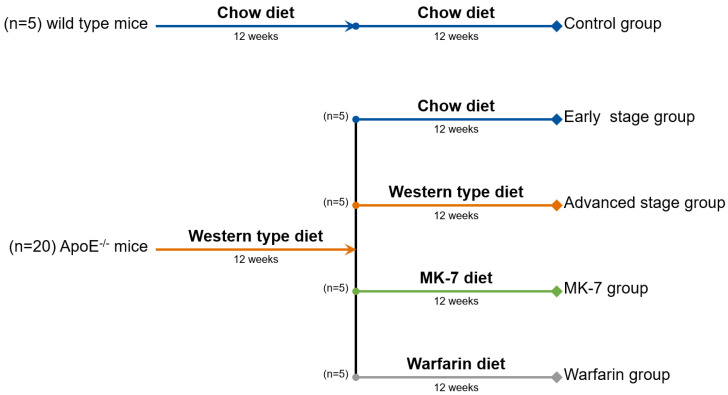
Feeding scheme: 20 ApoE^−/−^ mice started a Western-type diet for 12 weeks, in order to develop early stage plaque. At the end of the 12 weeks, 5 mice were switched to a regular chow feed (early stage group), 5 mice continued their Western type diet for the additional 12 weeks (advanced stage group), 5 mice were switched to a vitamin K deficient chow feed supplemented with MK-7 (100 μg/g of feed; MK-7 group), and the remaining 5 mice were switched to a vitamin K deficient chow supplemented with Warfarin (3 mg/g of feed) and Phylloquinone (1.5 mg/g of feed) for an additional 12 weeks (Warfarin group).

All of the mice used were male and were 6 weeks of age at the start of the trial. Upon arrival, five animals per experimental group were caged in order to ease the feeding scheme. Each mouse was fed for a total of 24 weeks prior to the measurements. The entire feeding scheme is illustrated in Figure 1.

At 6 weeks of age, all of the wild type mice (*n* = 5) started a regular chow diet (1324; Altromin, Lage, Germany), which was maintained for the entire length of the experiment (24 weeks).

At 6 weeks of age, ApoE^−/−^ mice (*n* = 20) started a Western-type diet with 0.15% cholesterol and 19.5% casein (Altromin, Lage, Germany) for a minimum of 12 weeks, in order to develop early stage plaque. For the early stage plaque group, the diet was switched to a regular chow feed (1324; Altromin, Lage, Germany), in order to slow the plaque development. For the advanced plaque group, the Western-type diet was continued throughout the additional 12 weeks.

To investigate the effect of vitamin K on plaque development, an additional MK-7 (kind gift of NattoPharma, Oslo, Norway) group and Warfarin (A4571-10G; Merck KGaA, Darmstadt, Germany) group were created. In the MK-7 group, after the original 12 weeks of the Western-type diet, the feed was switched to a vitamin K deficient chow diet (C 1020; Altromin, Lage, Germany) supplemented with MK-7 (100 μg/g of feed). The Warfarin group was switched to the same vitamin K deficient chow feed (C 1020; Altromin, Lage, Germany) supplemented with Warfarin (3 mg/g of feed) and Phylloquinone (1.5 mg/g of feed, V3501-10G; Merck KGaA, Darmstadt, Germany) [14].

During the acclimatization period, all of the mice maintained their respective feeding schemes.

Over the course of the study, three mice developed skin lesions suggestive of ulcerative dermatitis and received a topical Dexpanthenol ointment (i.e., one mouse from the control group, one from the MK-7 group, and one from the Warfarin group).

### 2.3. Na[^18^F]F Preparation

One Na[^18^F]F preparation was done for each scanning day.

The quaternary methyl ammonium (i.e., QMA) carbonate cartridges (186004540; Waters GmbH, Eschborn, Germany) were first conditioned with a 0.9% NaCl solution, then washed with sterile water. Afterwards, crude [^18^F]-fluoride (proton irradiated target water) was loaded onto the cartridge, washed with sterile water, and eluted with a 0.9% NaCl. This was used for the intravenous injections.

### 2.4. Na[^18^F]F PET/CT Measurements

All of the mice were imaged with a small animal PET/SPECT/CT system (i.e., Triumph^®^ II, Northridge Tri-Modality Imaging, Inc., Chatsworth, CA, USA), however only the PET and CT modalities were used for this study.

Under 1.5–2.5% isoflurane anesthesia in oxygen at 0.8 L/min, the lateral tail vein was injected with 50 μL of CT contrast agent (130-095-698; Viscover™) and 15 ± 2 MBq of Na[^18^F]F in a maximum total volume of 125 μL. After injection, the mice were placed on the scanner bed and the CT scan was initiated. The exposure settings used were as follows: 130 uA, 75 kVp, 230 ms exposure time, and 360° rotation with 720 views with an average of two frames for each view; the duration of the CT scans was ~15 min. A dynamic 1h PET scan was initiated at the end of the CT scan (i.e., ~25 min post injection). The CT had an axial field of view of 91.1 mm and a PET one of 112 mm. During the scans, the isoflurane concentration was adapted so as to achieve a respiratory rate between 75–50 breaths per minute.

One mouse from the early stage group was lost due to anesthesia overdose during the PET scan; therefore, this data was excluded from the final image analysis. However, tissues were used for the final validation stainings.

### 2.5. Image Processing and Analysis

The CT images were reconstructed using a Feldkamp filtered back projection reconstruction process to a voxel size of 0.154 × 0.154 × 0.154 mm^3^ in a 592 × 592 × 560 matrix. Using vendor software, the CT values were converted into Hounsfield units (HU) using the following formula

HU = 1000 × ((µ_t_ − µ_w_) ÷ µ_w_) (1)

where µ_w_ is the linear attenuation coefficient of the water and µ_t_ is the linear attenuation coefficient of the tissue.

The PET data were reconstructed using a 3D ordered-subset expectation maximization (i.e., OSEM-3D with three iterations and eight subsets) with a maximum a posteriori probability algorithm (30 iterations) into a 240 × 240 × 192 image matrix (resulting in final voxel dimensions of 0.25 × 0.25 × 0.597 mm^3^). PET normalization, CT attenuation correction, and CT scatter correction were applied to all of the PET reconstructions.

The PET images were automatically aligned to the CT using a custom-made transformation in PMOD software package version 3.13 (PMOD Technologies LLC, Zürich, Switzerland) from a capillary phantom. The co-registered PET/CT images were further used for the PET quantification. To avoid bone spillover, a bone auto-contour was defined as 5% of the activity (in kBq/cc) of the hottest registered voxel of the scan (i.e., ~450 kBq/cc) and was then masked. The manually drawn volumes of interest (VOIs) were placed at a distance of at least two voxels from the masked bone auto-contour. All of the VOI were placed on the axial view.

To calculate the blood pool background activity, a VOI, comprising of ~10 consecutive slices, was manually placed in the thoracic region of the inferior vena cava, encompassing the contrast enhanced lumen. The mean activity (in kBq/cc) and the average of the top 10 hottest voxels were recorded from this VOI.

A VOI for the left ventricle was created as follows: initially, the entire left ventricle (i.e., including the free ventricular wall, the interventricular septum, and the ventricular cavity) was manually isolated in a region spanning from the apex to the aortic root. Afterwards, an automatic isocontour was generated using the mean activity of the background (i.e., from the inferior vena cava VOI) as the minimal threshold.

The same technique was applied for the VOIs of the aortic root and aortic arch. Upon completion of all target VOIs, the average of the top 10 hottest voxels was recorded. All VOIs were drawn by a blinded member of our team, in order to exclude analysis bias.

In order to quantify the PET data and to correct for the blood compartment contribution, the maximum target-to-background ratio (TBRmax) was calculated using the following formula:

TBRmax = ((HotAverage(10)_target_ − HotAverage(10)_background_) ÷ HotAverage(10)_background_) (2)

where HotAverage(10)_target_ is the average of the top 10 hottest voxels recorded in the primary volumes of interest and HotAverage(10)_background_ is the average of the top 10 hottest voxels recorded in the inferior vena cava.

As only the Warfarin group presented modifications on the CT images, the data from only this group were also analyzed using PMOD software package version 3.13 (PMOD Technologies LLC, Zürich, Switzerland). The average background created by the blood pool and vessel wall was recorded by loading the aortic arch VOI that was created during the PET quantification and deleting the slices in which the radio-dense spots were visible (i.e., no thresholding was applied). One VOI, which included all spotty calcifications developed by one mouse, was serially drawn by hand in the axial slices, which contained no VOI for the background. Upon completion of all of the volumes of interest, the average of all of the voxels and the value of the hottest voxel were recorded. In order to quantify the CT data, the HU values of all volumes of interest were used.

### 2.6. Statistical Analysis

All of the variables are presented as data dots with a line and a value indicating the mean and error bars for the standard deviation. To calculate the statistical differences between multiple groups, one-way analysis of variance (one-way ANOVA) tests were applied. Additionally, the standard deviation of all of the variables was calculated and used alongside the average in the Results section. If the differences from this test exceeded the statistical significance threshold (i.e., *p* < 0.05), Tukey’s honestly significant difference test was performed for a post hoc analysis. Statistically significant results are indicated in charts, with stars suggesting a p-value lower than 0.05.

### 2.7. Organ Harvesting and Validation Staining Protocols

At the end of the scans, the mice were sacrificed by applying an isoflurane overdose, and the heart and aortic arch were collected using a well described protocol [15].

The aortic root was considered as the heart region where aortic leaflets are present. The aortic arch was considered to be the aortic region between the emergence of the ascending aorta from the heart and the third intercostal space; this sample also included the brachiocephalic artery and the emergences of the left common carotid and left subclavian artery.

All of the organ samples were formalin-fixed, paraffin-embedded, and sectioned with a thickness of 5 μm prior to staining using standard Hematoxylin eosin (HE) and von Kossa staining protocols.

## 3. Results

### 3.1. Validation Stainings

#### 3.1.1. HE Staining

**Figure 2:**
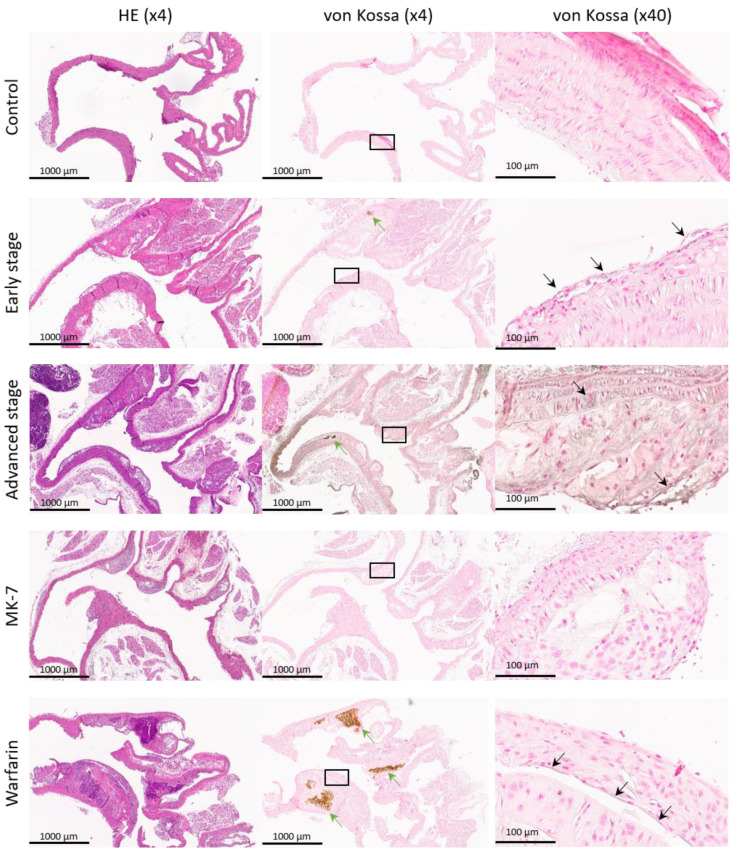
Validation stainings: example of an aortic arch Hematoxylin eosin (HE) and von Kossa stainings from each experimental group. The first image column is stained with HE followed by the next paraffin slice, which is stained with von Kossa (green arrows indicate calcifications larger than 50 μm). The von Kossa (×40) column contains representative enlargements with sites of microscopic calcification (black arrows indicate calcifications smaller than 50 μm).

All of the ApoE^−/−^ mice (i.e., the Warfarin, MK-7, advanced stage, and early stage groups) developed plaque, starting at the aortic root and then continuing along the aortic arch (Figure 2). Moreover, all of the mice from the Warfarin group presented several intense basic spots at different levels of the aortic arch—some in the aortic wall, while others in the wall of the brachiocephalic artery (Figure 2). The wild type control mice did not develop any plaque at the investigated levels (Figure 2).

#### 3.1.2. Von Kossa Staining

All of the mice from the Warfarin group exclusively presented dense mineralization spots in the aortic wall and the wall of the brachiocephalic artery, which co-localized with the intense basic spots from the HE staining (Figure 2). As these spots were present inside the plaque, this is suggestive of the pro-calcification potential of Warfarin.

### 3.2. CT Findings

**Figure 3:**
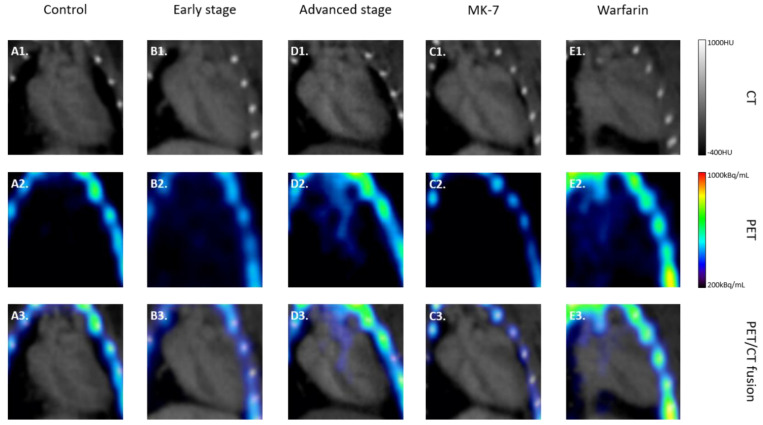
Na[^18^F]F PET/CT: examples of computed tomography (CT), positron emission tomography (PET), and PET/CT fusions of Na[^18^F]F uptake in the heart region of all of the experimental groups. Exemplary CT and PET are given in the first and second rows, respectively, while in the third row the fusions of the corresponding images are presented. All CT images are scaled between −400 HU and 1000 HU, while the PET images are between 200 kBq/mL and 1000 kBq/mL.

Examples of CT and PET/CT fusions of Na[^18^F]F uptake in the heart region of all of the experimental groups are illustrated in Figure 3.

The CT data from only the Warfarin group were analyzed, as in this group, radio-dense spots suggestive of spotty calcifications were observed in the proximal aorta. Each mouse presented at least two radio-dense spots in different regions of the proximal aorta. Table 1 presents the number and anatomical regions in which these spots were observed on the CT of each mouse from the Warfarin group.

**Figure 4:**
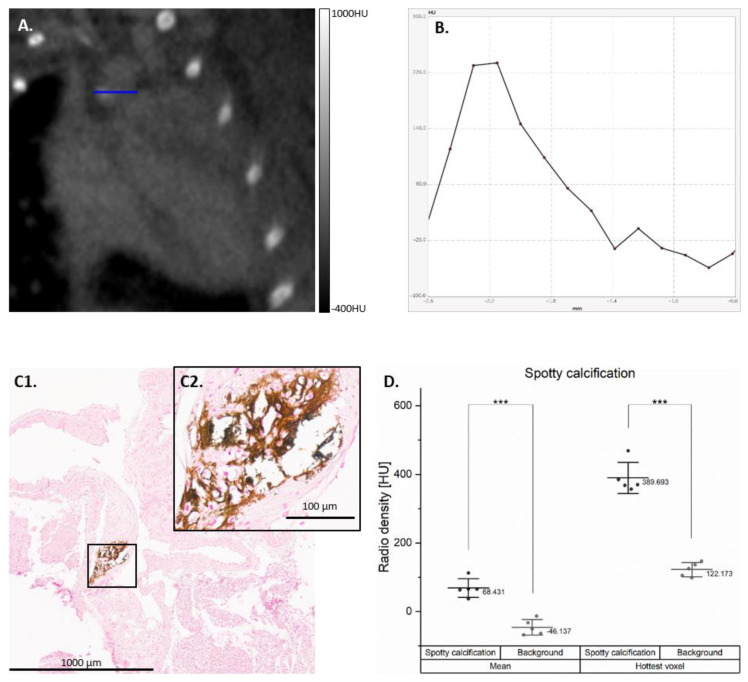
Spotty calcifications developed by the Warfarin group. Example of spotty calcification developed on the CT from the Warfarin group (**A**) with horizontal line profile (**B**). The spotty calcification was correlated with intense mineralization in the aortic arch in the von Koss staining (**C1**), with a ×40 enlargement (**C2**). The mean Hounsfield units (HU) value (averaged) and the hottest voxel (max) of the spotty calcifications are compared to the blood pool background created by the contrast agent (**D**); *** *p* < 0.001).

After a side by side comparison of the CT images with selected histological stainings (i.e., HE and von Kossa), all radio-dense spots corresponded with intense basic spots and dense mineralizations (Figure 4). Compared with the background created by the contrast agent in the blood, both the mean HU value of the calcification spots (*p* < 0.001) and the hottest voxel (*p* < 0.001) were significantly higher (Figure 4D).

### 3.3. Na[^18^F]F PET Findings

**Figure 5:**
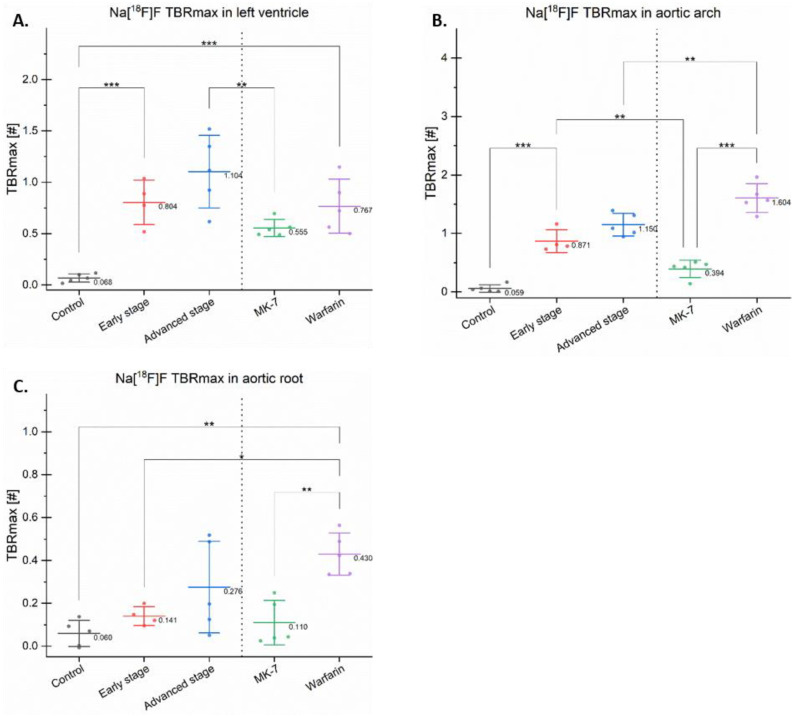
Maximum target-to-background ratios in different regions. The maximum target-to-background ratio (TBRmax) of Na[^18^F]F is presented as dots with a line and a value indicating the mean and error bars for the standard deviation in the left ventricle (**A**), aortic arch (**B**), and aortic root (**C**); * *p* < 0.05, ** *p* < 0.01, *** *p* < 0.001.

Examples of the PET and PET/CT fusions of Na[^18^F]F uptake in the heart region of all of the experimental groups are illustrated in Figure 3. Statistical analysis of all TBRmax values in the analyzed regions is presented in Figure 5. In all of the regions, the lowest TBRmax was found, as expected, in the control group followed by the MK-7 group (*p* = n.s.).

Na[^18^F]F TBRmax in all of the investigated regions seemed to reflect the duration of the Western-type diet (control vs. early stage vs. advanced stage). In the left ventricle and aortic arch, these increases reached statistical significance (*p* < 0.001), suggesting that these regions are more prone to develop micro-calcifications compared with the aortic root, which only includes the aortic leaflets.

The mice treated with MK-7 had a lower Na[^18^F]F TBRmax in all investigated regions compared with the Warfarin group. MK-7 reduced the Na[^18^F]F uptake, while Warfarin increased it, suggesting an analogue role in calcification.

In the aortic arch and left ventricle, the early, advanced stage, and Warfarin groups presented a high TBRmax compared with the control (*p* < 0.05), suggesting that all of the aforementioned groups developed calcifications (Figure 5A,B).

In the aortic root, significant differences were found in the control vs. Warfarin groups (*p* < 0.01), and in the early stage vs. Warfarin groups (*p* < 0.05). However, the differences between the control and both early and advanced stages were not significant.

In the aortic arch, the early stage had a significantly increased uptake compared with the MK-7 group (*p* < 0.01). Moreover, in this region, the Warfarin group had a higher TBRmax (*p* < 0.05) compared with the advanced stage group (Figure 5B). This increased uptake was due to the spotty calcifications developed by this new model, which is avid for Na[^18^F]F.

### 3.4. Skin Lesions Suggestive of Ulcerative Dermatitis

The images of the skin lesions developed by the three mice are available in the (Supplementary Material Figure S1). No correlation was observed between the diet and these skin lesions, as one was from the control group, one from the MK-7 group, and one from the Warfarin group. Post hoc, the PET data of these specific mice were double-checked; however, none of the mice had outlying TBRmax values within their respective groups. Therefore, the data of these mice were included in the statistical analysis.

Moreover, there was no abnormal bleeding observed in the lesions of the mouse from the Warfarin group, when compared with the other two animals.

## 4. Discussion

In this study, we have shown the potential ability of Na[^18^F]F to detect changes in diet in a uniform mouse model.

According to the maintenance phantom measurements, our small animal PET had a reconstructed resolution of ~1 mm^3^, while the mean cross-sectional plaque surface developed by ApoE^−/−^ mice (i.e., fed for 12 weeks with a high fat diet) was ~3000 μm^2^ [16]. Our targets were thus in the lower range of the PET detection; therefore, we decided to use relatively large VOIs so that multiple targets could be included. With a higher volume, the mean uptake is lowered by the blood pool activity, so we semi-quantitatively determined the uptake as a target of the background ratio of the hottest voxels (i.e., TBRmax), as suggested by other research groups in the field [17].

Our data suggest that Na[^18^F]F was able to discriminate between early and advanced stage plaque developed by ApoE^−/−^ mice fed a Western-type diet for 12 and 24 weeks, respectively. This trend may be due to the increase over time of the plaque calcium load [18]. Moreover, MK-7 supplementation seemed to have the lowest tracer uptake in the aortic arch and left ventricle of all of the other groups. In fact, uptakes in the MK-7 treatment and control groups were similar in all of the investigated regions. These results suggest that Na[^18^F]F is able to detect morphological changes induced by vitamin K treatment. One study showed that vitamin K reduced the plaque size and vascular (micro-) calcification levels in ApoE^−/−^ mice [16].

Recently, it has been shown that Na[^18^F]F is sensitive enough to reflect the changes of progressive exercise on atherosclerotic calcium deposits in ApoE^−/−^ mice [19], and also sustained vascular mineralization in an uremic mouse model [17]. Taken together with our data, it seems that Na[^18^F]F is able to distinguish between different stages of atherosclerosis, thus adding to the current trend for the introduction of Na[^18^F]F PET in clinical practice.

It was anticipated that the MK-7 group would have an increased uptake compared with the control, as these mice showed plaque in the histological analysis. However, the fact that this group had the lowest TBRmax compared with the other groups fed a Western-type diet suggests a protective role of MK-7 against micro-calcification formation, as revealed by the Na[^18^F]F uptake, which was already linked to the plaque calcification level [20].

Additionally, Na[^18^F]F revealed a difference between the continuation of the Western-type diet (i.e., advanced group) and the switch to a normal diet supplemented with vitamin K (i.e., MK-7 group). As C57BL/J mice consume on average 4 g of food per day [21], in our MK-7 group, each mouse received on average a daily supplementation of 400 μg vitamin K. This finding adds to the previously [5] described list of the beneficial effects of vitamin K for cardiovascular health, which, in combination with ongoing clinical trials, may favor the increase in the recommended daily dose of vitamin K or its introduction in clinical practice. Some clinical studies failed to observe a change in Na[^18^F]F uptake after 6 months [22] and 18 months [23] of MK-7 supplementation. This discrepancy between humans and mice may be explained by several changes in our preclinical study design, namely: mice have a higher metabolism, which is suggested to be 45 times faster in mice compared with humans [24]; moreover, taking into account the higher homogeneity of inbred mice, the variance is innately lower in preclinical studies compared with clinical trials.

Warfarin treatment induced the formation of calcified lesion in our mice. Previously, it was shown that when supplemented to a Western-type diet, it induced intimal plaque calcification in ApoE^−/−^ mice [14]. Moreover, this compound caused changes in the plaque morphology with features suggestive of plaque vulnerability [14].

An unexpected finding was the Warfarin-induced spotty calcifications detected by the CT. ApoE^−/−^ in a C57BL/6 background started to develop intra-plaque calcifications after 45 weeks of chow diet [25]. In our study, after 24 weeks of feeding, the Warfarin group developed spotty calcifications exclusively in the proximal aorta, despite extensive evaluation of all other regions. To our knowledge, this is the first detection via a small animal CT of the ectopic calcifications developed by Warfarin fed mice; moreover, the lesions developed were very similar to those observed in humans [8]. Similar lesions have been described in isolated aortas of 6-month old (i.e., 24 weeks) Ldlr^−/−^ mice scanned with a high resolution micro-CT suitable for small samples [26]. Other mouse models have developed vascular calcifications [27]; however, these mice require an additional mutation and they have a reduced life-span (i.e., Fetuin-A knock-out). On the other hand, there is no described reduced lifespan of the ApoE^−/−^ mice on Warfarin. In the present study, all of the mice from this group reached 6 months of age (i.e., 24 weeks of diet) with no apparent health problems. Moreover, in some of our still unpublished studies, the mice in this group reached 9 months of age (i.e., 36 weeks of diet), also with no apparent issues. To our knowledge, the only described side-effect is lethal bleeding, which may be kept in check by dietary supplementation with Phylloquinone [12].

No radio-dense spots were observed in the early and advanced stage groups, suggesting that the Western-type diet induced micro-calcified plaque, and only Warfarin supplementation induced spotty calcifications.

### 4.1. Limitations

The data generated by this study are mainly based on PET and CT analysis. Therefore, the assumptions that the different Na[^18^F]F uptakes reflect changes in the calcification micro-architecture are solely based on already published works. Irkle et al. [20] previously showed that this tracer is able to specifically detect active vascular calcifications in human plaque. Meanwhile, Hu et al. [18] recently reported that a similar correlation exists in ApoE^−/−^ mice at different ages. Therefore, in our study, no systematic qualitative assessments of plaque micro-calcification levels have been performed. As Na[^18^F]F has been shown to be able to detect calcification sites invisible by regular histological stainings [10,20], additional investigations that have been performed for other models [14,26,28] are still required (e.g., micro-autoradiography, atomic absorption-spectrometry, or energy-dispersive X-ray spectroscopy).

Another limitation of the presented study and mouse model is the extended feeding scheme, which prevented a post hoc repeat of this study. Additionally, the long feeding scheme combined with the C57BL/6 background led to some mice developing skin lesions suggestive of ulcerative dermatitis, for which a Dexpanthenol ointment (Bepanthen^®^ from Bayer Vital GmbH, Leverkusen, Germany) was applied to ease scratching. However, there was no described influence of Dexpanthenol topical ointment on atherosclerotic development.

Lastly, it is worth mentioning that the term “spotty calcification” was used to describe the radio-dense spots in the aortic arches of the Warfarin group, however there is no clear-cut definition of this term in preclinical research [29].

### 4.2. Future Prospects

Na[^18^F]F has shown its ability to target vulnerable micro-calcified plaque in many independent studies [5]. Our findings also point to its utility in monitoring treatment and disease progression. However, further clinical studies are also necessary to show its potential in human plaque, as mice develop morphologically different plaque [30]. This data opens the path for future clinical studies to be able to debate the use of Na[^18^F]F PET in micro-calcified plaque monitoring. The same is true for our vitamin K data, which will come to the aid of future studies that want to investigate the benefits of this complex on vascular health.

As spotty calcifications detected on CTs are still used in the clinical diagnosis of vulnerable plaque, our new Warfarin model may be used to first assess the efficacy of novel therapeutic approaches in a preclinical setup. We do not expect excessive bleeding during the surgery of these mice, as the animal with skin lesions did not present any abnormal bleeding in comparison with the other two mice with the same condition.

## 5. Conclusions

In this study, Na[^18^F]F PET showed potential to monitor plaque progression and treatment. This tracer also seemed to be able to detect the beneficial effects of vitamin K on cardiovascular micro-calcification, when compared with the continuation of a Western-type diet. Additionally, we describe a novel mouse model, which spontaneously develops spotty calcifications, detected by small animal CT, exclusively in the proximal aorta, with no apparent life-span reduction.

## Figures and Tables

**Table 1:** Number and anatomical regions of the radio-dense spots observed in the Warfarin group.

Mouse NO.	Aortic Arch	Brachiocephalic Artery	Left Carotid Artery	Total ^1^
1	2	1	0	3
2	1	1	1	3
3	0	1	1	2
4	2	1	0	3
5	2	0	0	2

^1^ Total number of spotty calcifications developed by each mouse.

## Data Availability

Data available in a publicly accessible repository. The data presented in this study are openly available in Zenodo at DOI:10.5281/zenodo.4478936.

## Supplementary Materials

The following are available online at https://www.mdpi.com/2073-4409/10/2/275/s1: Figure S1: Ulcerative dermatitis.
