# Supplementary material for: Sodium [18F]Fluoride PET Can Efficiently Monitor In Vivo Atherosclerotic Plaque Calcification Progression and Treatment
Source: Cells. 2021 Jan 30;10(2):275. doi: 10.3390/cells10020275 (PMC7911917; doi:10.3390/cells10020275)
Supplement: Supplementary file 1 [file cells-10-00275-s001.pdf]

Supplementary Material:

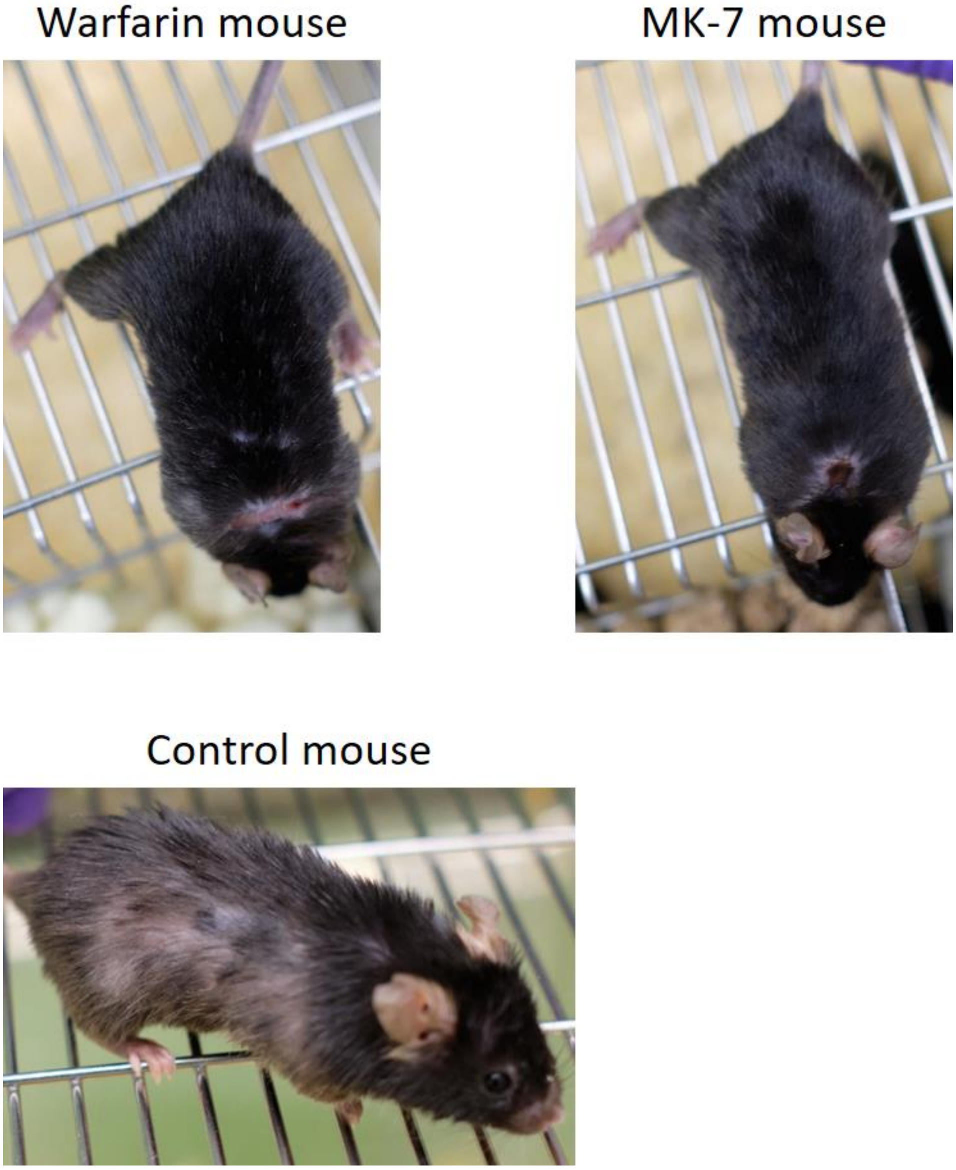

Figure S1. Ulcerative dermatitis. Images of skin lesions suggestive of ulcerative dermatitis developed by the mice from the Warfarin, MK-7, and control group. There was no abnormal bleeding observed in the lesions of the mouse from the Warfarin group, when compared with the other two animals.
